# The Central Role of Salivary Metalloproteases in Host Acquired Resistance to Tick Feeding

**DOI:** 10.3389/fcimb.2020.563349

**Published:** 2020-11-18

**Authors:** Jan Perner, Dominic Helm, Per Haberkant, Tereza Hatalova, Sara Kropackova, Jose M. Ribeiro, Petr Kopacek

**Affiliations:** ^1^Institute of Parasitology, Biology Centre, Czech Academy of Sciences, Ceske Budejovice, Czechia; ^2^Proteomics Core Facility, The European Molecular Biology Laboratory (EMBL), Heidelberg, Germany; ^3^Laboratory of Malaria and Vector Research, National Institute of Allergy and Infectious Diseases, Bethesda, MD, United States

**Keywords:** ticks, acquired resistance, antigen, immunoprecipitation, vaccine, *Ixodes ricinus*, metalloprotease

## Abstract

During feeding on vertebrate hosts, ticks secrete saliva composed of a rich cocktail of bioactive molecules modulating host immune responses. Although most of the proteinaceous fraction of tick saliva is of little immunogenicity, repeated feeding of ticks on mammalian hosts may lead to impairment of tick feeding, preventing full engorgement. Here, we challenged rabbits with repeated feeding of both *Ixodes ricinus* nymphs and adults and observed the formation of specific antibodies against several tick salivary proteins. Repeated feeding of both *I. ricinus* stages led to a gradual decrease in engorged weights. To identify the salivary antigens, isolated immunoglobulins from repeatedly infested rabbits were utilized for a protein pull-down from the saliva of pilocarpine-treated ticks. Eluted antigens were first identified by peptide mass fingerprinting with the aid of available *I. ricinus* salivary gland transcriptomes originating from early phases of tick feeding. To increase the authenticity of immunogens identified, we also performed, for the first time, *de novo* assembly of the sialome from *I. ricinus* females fed for six days, a timepoint used for pilocarpine-salivation. The most dominant *I. ricinus* salivary immunogens identified in our study were zinc-dependent metalloproteases of three different families. To corroborate the role of metalloproteases at the tick/host interface, we fed ticks micro-injected with a zinc metalloprotease inhibitor, phosphoramidon, on a rabbit. These ticks clearly failed to initiate feeding and to engorge. However, neither feeding to ticks immune blood of repeatedly infested rabbits, nor phosphoramidon injection into ticks, prevented their engorgement when fed *in vitro* on an artificial membrane system. These data show that Zn metalloproteases play a decisive role in the success of tick feeding, mediated by complex molecular interactions between the host immune, inflammatory, and hemostatic processes, which are absent in *in vitro* feeding. This basic concept warrants further investigation and reconsideration of the current strategies towards the development of an effective “anti-tick” vaccine.

## Introduction

Ticks and tick-borne diseases (TBD) represent a growing global burden for both human and animal health (de la Fuente et al., 2008) and are a major constraint for the improvement of livestock industries, particularly in developing countries (Peter et al., 2005). Host immunity-mediated rejection of ticks has potential in the search for a vaccine that offers an effective and environmentally sound approach for controlling ticks and TBDs (de la Fuente et al., 2016).

Ticks salivate proteinaceous saliva into the host during the course of feeding (Francischetti et al., 2009). The individual protein components are secreted into a feeding site at functional concentrations and with adequate affinities for host targets to establish biomolecular associations at the feeding site, thereby modulating host intrinsic hemostatic processes and defence responses (Mans, 2019). To escape immune recognition by the host, tick salivary proteins have evolved low immunogenicity (Chmelar et al., 2016), leading to low or no tick rejection reactions when *Ixodes ricinus* ticks feed on their natural hosts such as mice, voles, or passerine birds (Dizij and Kurtenbach, 1995; Dusbábek et al., 1995; Heylen et al., 2010). In contrast, tick saliva induces an antibody-mediated response in distinct less adapted hosts (Brossard and Wikel, 2005).

The production of anti-salivary gland-specific antibodies against *Amblyomma variegatum* and *I. ricinus* ticks was reported in rabbits (Jongejan et al., 1989; Schorderet and Brossard, 1993). Furthermore, sera from sheep that were exposed to a repeated infestation of *Amblyomma americanum* ticks were shown to recognize multiple tick antigens (Barriga et al., 1991). The presence of specific anti-tick antibodies has been correlated with the inability of ticks to feed and/or reproduce, leading to the concept of acquired resistance to ticks in sensitive host species (Bowessidjaou et al., 1977). The presence of host antibodies in the tick’s blood meal might thus be a decisive defence factor preventing tick infestations.

The cellular fraction of host immunity is also clearly involved in the phenomenon of acquired resistance to ticks. In fact, cellular infiltration into the tick feeding site was described in the original seminal paper (Trager, 1939) showing that ticks fed on resistant animals were pale in colour due to a higher leukocyte content in their gut. It was established that repeated tick infestation on guinea pigs is characterized by a large accumulation of eosinophils and basophils in the dermis and epidermis (Allen, 1973). Similarly in mice, basophils are recruited to the tick feeding site during the second, but rarely the first, infestation (Wada et al., 2010). The indispensable role of basophils in acquired resistance to ticks was demonstrated convincingly by diminishing the manifestation of acquired resistance in basophil-ablated mice (Wada et al., 2010).

In the more-than-20-year-old review (Willadsen and Jongejan, 1999), it was stated: “Disappointingly, little progress has been made in the identification of the protective antigens of naturally acquired immunity.” A few studies have addressed this challenge in various tick species since then by using yeast surface display (Schuijt et al., 2011) or gel-based proteomics (Garcia et al., 2017). To fill the knowledge gap as to what are the *bona fide* immunogens introduced into the host by tick feeding, we exploited a laboratory model of repetitive infestations of *I. ricinus* adult females and nymphs on rabbits to induce the production of antibodies. Using pull-down enrichment of proteins present in *I. ricinus* saliva *via* their specific binding to immunoglobulins from resistant rabbits, and following differential proteomic analysis, we identified natural tick antigens against which, antibodies are formed in the acquired immunity of rabbits. Our data suggest that zinc-dependent metalloproteases in tick saliva play the most prominent role in mounting an antibody response in resistant rabbits.

## Material and Methods

### Tick Colony Maintenance and Natural Feeding on a Rabbit

*I. ricinus* ticks (nymphs and adults) were collected by flagging in the forest near České Budějovice, Czech Republic. Ticks were kept at 24°C and 95% humidity under a 15:9 h day/night regime. Repeated feeding of adult *I. ricinus* ticks was performed by placing 50 freshly collected females into two chambers (25 each with an equal number of males) on the opposing sides of the shaven back of a laboratory rabbit (Hy-Plus strain) and ticks were allowed to feed naturally until replete. The feeding was repeated three times at 2–3 week intervals, and engorged females were weighed after each feeding. Feeding of *I. ricinus* nymphs on a laboratory rabbit was performed in total, eight times: 4 x 2 subsequent feedings (10 days overall) followed by a resting interval of 2–3 weeks (1st, 2nd, 3rd, or 4th nymphal infestation). For each feeding, a total number of 500 nymphs were equally distributed into two chambers on the opposing sides of the rabbit and allowed to feed till replete. Fifty representative fully fed nymphs were weighed per feeding.

All laboratory animals were treated in accordance with the Animal Protection Law of the Czech Republic No. 246/1992 Sb., ethics approval No. 25/2018. The study was approved by the Institute of Parasitology, Biology Centre of the Czech Academy of Sciences (CAS) and Central Committee for Animal Welfare, Czech Republic (protocol no. 1/2015).

### Membrane Tick Feeding

Membrane feeding of ticks was performed according to the original protocol (Krober and Guerin, 2007) in a 6-well plate format kept at 37°C. Bloods from repeatedly infested and naive rabbits were collected in 50 ml conical centrifuge tubes and manually defibrinated using glass beads (diameter 4 mm). Fifteen females were placed into each feeding unit lined with a thin (80–120 μm) silicone membrane, previously pre-treated with a bovine hair extract in dichloromethane (0.5 mg of low volatile lipids). After 24 h, unattached or dead females were removed and an equal number of males were added to the feeding unit containing attached females. Diets were exchanged in a 12 h regime, with concomitant addition of 1 mM adenosine triphosphate (ATP) and gentamicin (5 μg/ml).

### Micro-Injection of Unfed *I. ricinus* Females

Phosphoramidon [*N*-(α-rhamnopyranosyl-oxyhydroxyphosphinyl)-Leu-Trp] (Merck; Sigma-Aldrich 525276**)**, a competitive inhibitor of several soluble zinc metalloproteases (Kitagishi and Hiromi, 1984), was solubilized in PBS and 300 nl were micro-injected in a concentration series ranging from 5–50 µg/µl, through the coxa of the third pair of legs into the hemocoel of each unfed adult *I. ricinus* female using a Nanoinject II (Drummond). Next day, ticks were allowed to feed naturally on a rabbit or were fed *in vitro* on a membrane (see above). The feeding ability of ticks was monitored, and their post-engorgement weights were determined.

### Salivary Gland Transcriptome Assembly

Five pairs of salivary glands were dissected from adult *I. ricinus* females fed for 6 days on a naïve rabbit. These were homogenized using a 29G needle syringe and RNA was extracted using Nucleospin RNA (Macherey-Nagel). The RNA extract was sent to Novogene for library preparation and NovaSeq 600 M (Illumina) sequencing. Paired end unstranded sequencing (PE 150) generated 39,825,389 clean reads with a Q30 of 95%. Transcriptome assembly and coding sequence extraction were carried out as described previously (Ribeiro et al., 2014). Briefly, reads were stripped of their contaminating primers, and bases with equal values <20 were trimmed. Clean reads were assembled using the Abyss (Birol et al., 2009) and Trinity (Grabherr et al., 2011) assemblers. These assemblies were merged using a parallelized pipeline of blastn and cap3 assembler (Huang and Madan, 1999) as described previously (Karim et al., 2011). All open reading frames larger than 200 nucleotides were extracted and those matching known proteins or having a signal peptide were retained. The resulting peptide and coding sequences were mapped to a hyperlinked spreadsheet, including blastp and rpsblast matches to several databases, as well as an indication of the signal peptide (Nielsen et al., 1999), transmembrane domains (Sonnhammer et al., 1998) and O-galactosylation sites (Hansen et al., 1998). The sequence data are available in GenBank as BioProject: 589581 and a hyperlinked excel sheet is available to download at https://proj-bip-prod-publicread.s3.amazonaws.com/transcriptome/Perner/Ixric/Perner-Ir2019.zip (further referred to as Source data 1), which also provides semi-quantitative data on transcript expression. Column AR in the Source data 1 spreadsheet indicates a Y if the new sequence is at least 2% different from the public one, or if it extends the length of a known sequence. The Y actually represents 3 classes: (a) novel sequences, if less than 95% identical to a known sequence, (b) similar sequences, possibly due to alleles (95–98% identity and full or at least 90% coverage, (c) extensions, with 98–100% id and longer new sequences as indicated in column AQ of the Source data 1 spreadsheet.

### Immunoprecipitation

Saliva was collected from one hundred *I. ricinus* adult females fed on naïve rabbits for 6 days and they were then manually detached and removed. Salivation was induced by application of 1.2 µl of pilocarpine (5 mg dissolved in 100 µl of 100% ethanol) onto the tick scutum (Tatchell, 1967). Saliva was collected into 10-µl capillaries for 2 h in a wet chamber kept at 30°C. IgG fractions were obtained from the rabbit sera by caprylic acid precipitation of non-IgG proteins (Russo et al., 1983) and dialysed against 50 mM Tris, pH 8.0, 150 mM NaCl. A volume of 125 µl of Protein A Mag Sepharose bead slurry (GE Healthcare) was equilibrated with 1 ml of binding buffer: 50 mM Tris, pH 8.0, 150 mM NaCl. Immunoglobulins (82.5 µg) were then added to equilibrated beads and incubated for 1 h at 7°C. Beads were washed three times with binding buffer and then saliva was added to the Ig-loaded beads. These were incubated over-night at 7°C in a head-over-head rotator. Beads were washed five times with binding buffer and antigens were eluted with 0.2% SDS, 0.1% Tween-20, 50 mM Tris-HCl, pH  =  8.0, 150 mM NaCl incubated for 7 min at 25°C, shaking at 1,000 rpm (Antrobus and Borner, 2011). The eluates were separated by non-reducing SDS-PAGE in 4–12% Bis Tris Plus gel (Life Technologies) and stained with Coomassie Brilliant Blue.

### Sample Preparation for Mass Spectrometry

Coomassie-stained bands were excised, chopped into small pieces and transferred to 0.5-ml Eppendorf tubes. For all following steps, buffers were exchanged in two consecutive 15-min incubation steps of gel pieces with 200 µl of acetonitrile (ACN), with the ACN being removed after each step. Proteins were reduced by the addition of 200 µl of a 10 mM DTT solution in 100 mM ammonium bicarbonate (AmBiC, Sigma Aldrich, A6141) and incubated at 56°C for 30 min. Proteins were alkylated by the addition of 200 µl of 55 mM iodoacetamide (IAA) in 100 mM AmBiC and incubated for 20 min in the dark. A 0.1 µg/µl stock solution of trypsin (Promega, V511A) in trypsin resuspension buffer (Promega, V542A) was diluted with ice-cold 50 mM AmBiC buffer to achieve a final concentration of 1 ng/µl. 50 µl were then added to the gel pieces, which were incubated first for 30 min on ice and then overnight at 37°C. Gel pieces were sonicated for 15 min, spun down and the supernatant was transferred into a glass vial (VDS Optilab, 93908556). Remaining gel pieces were washed with 50 µl of an aqueous solution of 50% ACN and 1% formic acid and sonicated for 15 min. The combined supernatants were dried in a speedvac and reconstituted in 10 µl of an aqueous solution of 0.1% (v/v) formic acid.

### LC-MS/MS

Briefly, peptides were separated using an UltiMate 3000 RSLC (Thermo Scientific) equipped with a trapping cartridge (Precolumn; C18 PepMap 100, 5 lm, 300 lm i.d. × 5 mm, 100 A°) and an analytical column (Waters nanoEase HSS C18 T3, 75 um × 25 cm, 1.8 µm, 100 A°). Solvent A: aqueous 0.1% formic acid; Solvent B: 0.1% formic acid in acetonitrile (all solvents were of LC-MS grade). Peptides were loaded onto the trapping cartridge using solvent A for 3 min at a flowrate of 30 µl/min. Peptides were separated on the analytical column at a constant flowrate of 0.3 µl/min applying a 30 min gradient of 2–25% of solvent B in A, followed by an increase to 85% B. Peptides were directly analyzed in positive ion mode applied with a spray voltage of 2.2 kV and a capillary temperature of 275°C using a Nanospray-Flex ion source and a Pico-Tip Emitter 360 lm OD × 20 lm ID; 10 lm tip (New Objective). The MS spectra with a mass range of 350–1.500 m/z were acquired using a resolution of 120.000 (maximum injection time of 100 ms). Fragmentation was triggered by HCD in fixed collision energy mode with fixed collision energy of 30% for peaks with a charge state of 2–6 on the MS scan and a 5-second exclusion window. MS2 spectra were acquired by the Ion Trap with a rapid ion trap scan rate and a max injection time of 35 ms. The mass spectrometry proteomics data have been deposited to the ProteomeXchange Consortium *via* the PRIDE (Perez-Riverol et al., 2019) partner repository with the dataset identifier PXD021370.

### Data Analysis

Acquired data were processed using MaxQuant 1.6.7.0 with cDNA databases based on transcriptomics data. Apart from the default settings, the following modifications were considered: Carbamidomethyl (C) (fixed modification), Acetyl (N-term), and Oxidation (M) (variable modifications). Gel slices were considered as fractions of the naïve or immunized samples. Proteins were identified by a minimum of four assigned unique peptides and, with zero assignment in the naïve sample, are shown in **Table 1**. Only sequence coverages of more than 2% and Q values (representing the probability that the protein is a false hit) that equal zero were considered. Full proteomics assignment data are shown in the excel spreadsheet available as **Supplementary Data File**.

### Statistical Analysis

Data on tick weights failed to meet the criteria for normal data distribution according to the Shapiro-Wilk normality test. Individual datasets were, therefore, analyzed by the unpaired non-parametric Mann-Whitney test. Individual statistical significances are stated in the Figure legends.

## Results

### Repeated *Ixodes ricinus* Feeding Induces Acquired Resistance in Rabbits

**Figure 1:**
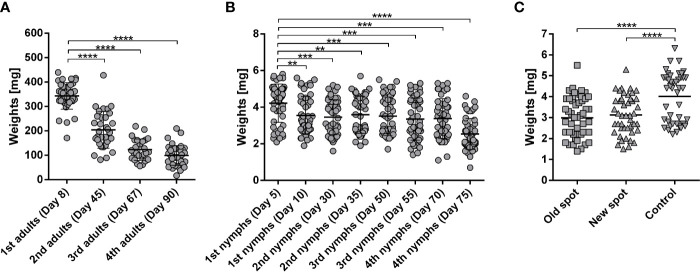
**(A)** Weights of *Ixodes ricinus* adults repeatedly fed on one rabbit. *I. ricinus* adult females were fed on a laboratory rabbit in subsequent feedings with 2–3-week intervals between feedings. Adult females were placed in two chambers on opposing sides of the rabbit (25 ticks into each chamber); n ≥ 30; Days indicate the date of detachment of respective groups after attachment of the first ticks. **(B)** Weights of *I. ricinus* nymphs repeatedly fed on one rabbit. *I. ricinus* nymphs were fed on a laboratory rabbit in two subsequent feedings (10 days overall) and with 2–3-week intervals between feedings. Nymphs were placed in two chambers on opposing sides of the rabbit (125 nymphs into each chamber). Two subsequent feedings (1^st^, 2^nd^, 3^rd^, or 4^th^ nymphs) totaled each 500 nymphs. Days indicate the date of detachment of the respective groups after attachment of first ticks. Fifty representative fully fed nymphs were weighed per feeding. Note, the clusters above and below the average weight represent weights of female and male nymphs, respectively (Dusbabek, 1996). **(C)** Weights of *I. ricinus* nymphs fed on a repeatedly adult-infested rabbit. *I. ricinus* nymphs were fed on a laboratory rabbit previously fed on by *I. ricinus* adults. Nymphs were placed on a rabbit two weeks after the last *I. ricinus* adult feeding (Day 90). Nymphs were placed either on a spot of previous feeding (old) or the feeding chamber was moved to a previously un-infested spot on the same rabbit (new). Nymphs fed on a naive rabbit were used as a control. Mean + SEM are shown, n ≥40. Asterisks indicate statistical significance, ****p < 0.0001, ***p < 0.001, **p < 0.01, when compared to tick weights after first feeding.

We subjected laboratory rabbits to repeated feeding of *Ixodes ricinus* adults and nymphs. After initial feeding, any following feeding of adult females led to significantly decreased engorged weights (**Figure 1A**). Similarly, the immediate weight reduction of nymphs following feeding of the first cohort was observed with a gradual and continuous decrease in nymphal weights until the 4^th^ infestation (**Figure 1B**). To verify if the acquired resistance to adult ticks would also lead to resistance against nymphal stages, we added *I. ricinus* nymphs to a rabbit with previously developed resistance to *I. ricinus* adults. Nymphs were placed onto the same spot where adults had previously fed, and also onto a new unexposed spot on the rabbit’s back (**Figure 1C**). Nymphs gained significantly lower engorged weights compared to control nymphs fed on a naïve rabbit, demonstrating cross-reactivity between adult and nymphal antigens and cross-protection between adult and nymphal *I. ricinus* ticks (**Figure 1C**). There was no significant difference between ticks placed on the old and new spot of the rabbit, indicating a systemic response to the tick feeding rather than local rejection (**Figure 1C**).

### Immunoprecipitation of Antigens From Tick Saliva Using Antibodies Developed Under Naturally Acquired Immunity

**Figure 2:**
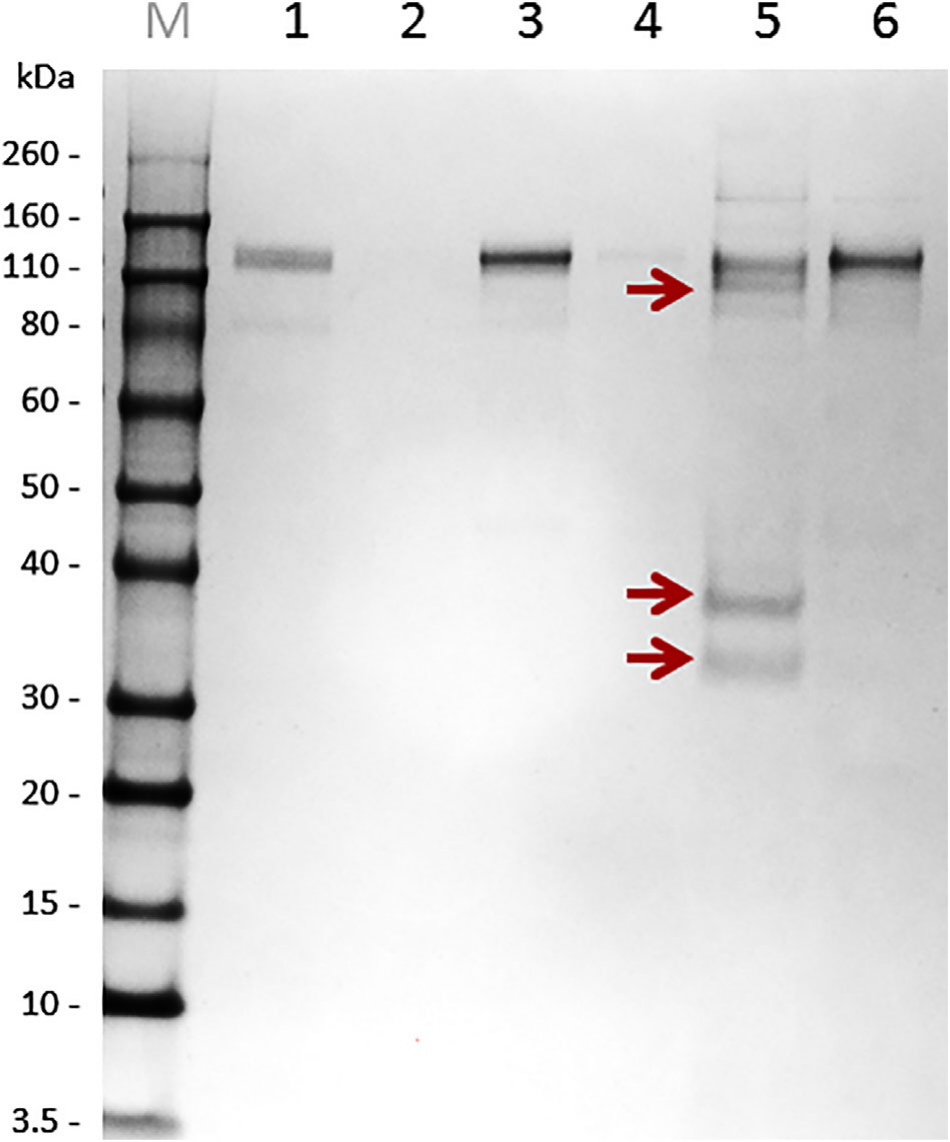
Non-reducing SDS-PAGE separation of immunoprecipitated salivary antigens using protein A paramagnetic beads. Immunoglobulins (Ig’s) from both immunized (repeated feeding) and naïve rabbit sera were immobilized on Protein A magnetic beads. These immunoglobulin-loaded beads were used for antigen fishing in pilocarpine-induced saliva of *I. ricinus* females fed on a rabbit for six days. Bound antigens to immunoglobulins from immunized and naïve rabbits were eluted and subjected to SDS-PAGE. M – Novex Sharp Marker, 1 – immunoglobulin fraction from “immunised” rabbits, 2 – SN after bead pelleting (indicates immobilization of “immunised” Ig’s on Protein A beads), 3 – immunoglobulin fraction from naïve rabbits, 4 – SN after bead pelleting (indicates immobilization of naive Ig’s on Protein A beads), 5 – elution fraction from “immune” beads, 6 – elution fraction from “naïve” beads. Bands from experimental group (red arrows) and corresponding areas from the control gel control were sliced out the gel and subjected to proteomic analysis.

Tick secretory material is derived predominantly from salivary glands but can also contain components from midgut regurgitate (Brown, 1988). To select the source material for pull-down, we inspected the background avidity of naïve rabbit immunoglobulins against midgut homogenates, salivary gland homogenates, and saliva (**Supplementary Figures S1–3**). While immunoglobulins from naïve rabbits markedly cross-reacted with proteins of the tick midgut and salivary gland homogenates (**Supplementary Figures S1, 2**, respectively), reduced immuno-detection was found in tick pilocarpine-induced saliva using immunoglobulins from naïve rabbits (**Supplementary Figure S3**). Therefore, for identification of immunogens, we used saliva of *I. ricinus* ticks, rather than a tissue homogenate. Isolated immunoglobulins from sera of immunized or naïve rabbits were immobilized on paramagnetic ProteinA beads, and target antigens were pulled-down from the saliva of adult *I. ricinus* females. To prevent co-elution (leaking) of immunoglobulins with the antigens, we used mild elution conditions for retrieval of a pure, yet possibly not exhaustive, set of antigens. The elution fraction from immunoglobulins of the repeatedly infested rabbit clearly contained specific antigens that were not present in the elution fraction from immunoglobulins of a naive rabbit (**Figure 2**). The elution fractions were separated by SDS-PAGE and the area around the visible bands were excised for proteomic identification, together with the corresponding gel areas in the lane from the naïve rabbit control.

### Tick Antigens Recognized by Naturally Acquired Immunity Are Mostly Secreted Metalloproteases

Proteins were, by default, identified by assignment of obtained peptides in available *I. ricinus* transcriptomes from the early phase of feeding. Existing transcriptomes of *I. ricinus* salivary glands, however, so far have covered a transcript repertoire of salivary glands only during the initial three days of tick feeding (Schwarz et al., 2013; Kotsyfakis et al., 2015; Perner et al., 2018). In order to fine-tune the protein identification of salivary gland antigens at the timepoint of saliva collection, we assembled a new transcriptome from salivary glands of adult *I. ricinus* females fed for six days. Additionally, we have identified 19,559 novel or extended transcripts (see *Material and Methods* section for the filtering criteria). The full list of assembled contigs, their sequences and expression values are accessible through the Source data 1, a hyper-linked excel sheet (see *Material and Methods* for a download link). Using this new transcriptome as a search database for peptide assignments (BioProject: 589581), we have identified only one unique antigen-encoding transcript on top of those identified from already existing transcriptomes (**Table 1**). Out of ten identified antigens, six are enzymes, one is a binding protein, and three are proteins of unknown function (containing a distant domain similarity with the catalytic domain of phosphoinositide-specific phospholipase C-like phosphodiesterases) (**Table 1**). Among the identified salivary antigens, the MA clan metalloproteases of the GluZincin superfamily (based on MEROPS nomenclature), and the zinc-dependent metallopeptidases, which contain the HEXXH motif as part of their active site, clearly dominate. These mainly include the M13 family neprilysin-like metalloprotease, the M2 family angiotensin converting enzyme-like dipeptidyl carboxypeptidase (kininase), and the M1 family aminopeptidase N, venom metalloprotease 3, and Metis5 (venom antarease-like) metalloprotease. Additionally, a non-proteolytic enzyme identified among the enriched antigens was the ATP diphosphohydrolase (apyrase), a frequently reported constituent of tick saliva (Mans, 2016). The full list of identified antigens and numbers of assigned peptides is available as **Supplementary Data File**. To gain an insight into expression dynamics throughout tick feeding, we inspected the transcription expression profiles of those transcripts that were present in our new 6 days fed SG library and compared their expression patterns in SG RNAseq databases from earlier timepoints of feeding (Source data 1 spreadsheet, columns ER-EZ). Most of the antigen transcripts were apparently present in the salivary glands during the early phases of feeding. In fact, most were highly abundant transcripts and their levels decreased only slightly as feeding progressed (**Table 1**). A unique transcript, present only in the new 6 days fed SG library, was the venom metalloprotease 3. Based on software predictions, most of the identified antigens are secretory proteins except aminopeptidase N that lacks the signal peptide (**Table 1**). The antigens identified were predicted to be frequently post-translationally modified, bearing O-glycosylation at Ser and Thr residues, and/or N-glycosylation at Asn residues (**Table 1**). This is in line with the recent corroboration that the presence of a glycan moiety on salivary proteins is critical for eliciting tick rejection by a host, a guinea pig in that case (Narasimhan et al., 2020). Of note, a clear motif ANGD**Y**DDWQ, found in the sequence of aminopeptidase N and apolipophorin, indicates sulfation of the tyrosine residue of the enzyme (**Table 1**), a key post-translational modification that determines the capacity of tick salivary proteins to inhibit host clotting enzymes (Thompson et al., 2017).

**Figure 3:**
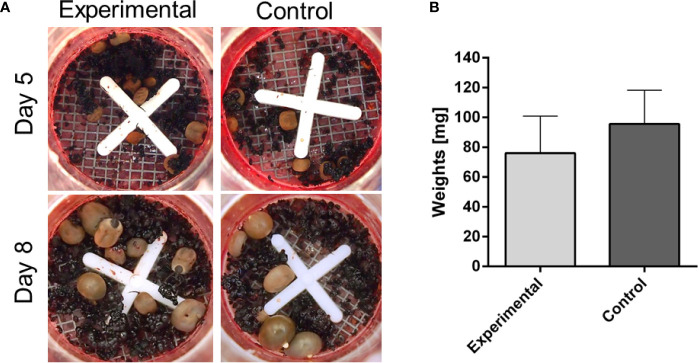
Membrane feeding of *Ixodes ricinus* adults on blood from infested rabbit. *I. ricinus* adults were fed on reconstituted rabbit blood (haematocrit 60%) either from a previously infested rabbit, by repeated adult *I. ricinus* feeding (experimental), or from a naïve rabbit (control). **(A)** Pictures of feeding units during the course of artificial feeding. **(B)** Weights of dropped-off females and females removed on Day 10. Bars represent a mean and SEM, n ≥6.

### Tick Membrane Feeding of Immunized Rabbit Blood Did Not Lead to Decreased Engorged Weights

To examine whether the decreased weights of ticks fed on repeatedly infested rabbits is caused by the presence of “toxic” immunoglobulins in the blood, we performed the following *in vitro* feeding experiment. Ticks were fed artificially on blood collected from repeatedly infested rabbits and naïve rabbits. The results clearly show that both groups achieved comparable engorged weights irrespective of the presence of anti-tick immunoglobulins (**Figure 3**). This observation indicates that integration of antibodies in acquired resistance to ticks is not an independent cause but is likely linked to other fractions of host immunity absent in the artificial membrane feeding. This is in line with a previous observation that the humoral response of the immunized animals alone did not affect tick feeding (Knorr et al., 2018). Also, this result rather negates the expectations of using this *in vitro* feeding platform as a valuable tool for screening anti-tick sera from vaccinated animals, as previously proposed (de la Fuente et al., 2016).

**Table 1:** Identification and characterization of tick *I. ricinus* salivary antigens.

Protein name^a^	**Transcript IDs**^**b**^**GenBank** SG 6D transcriptome	Unique peptides^c^ Immunised	Sequence coverage^d^ (unique)[%]	SignalP^e^	PTM^f^ predictions	Expression in SG^g^[FPKM values]			
						1D	2D	3D	6D
Neprilysin-like peptidase	GADI01003586 **IrSigP-237310_FR2_13-780**	**13**	14.5 (14.5)	**Y**	**O5**_**N2**_Y0	**156**	**92**	**60**	**42**
Apolipophorin	GANP01007796 GANP01012862	**7**	30.7 (2.7)	**Y**	**O4**_**N1**_**Y1**	nd	nd	nd	nd
ACE-like (Kininase)	**IrSigP-1798_FR1_163-82**	**7**	45.9 (10)	**Y**	O0_**N2**_Y0	**957**	**971**	**1,406**	**448**
Aminopeptidase N	GADI01006478 **234210_FR3_1-970**;	**5**	11.4 (9.7)	N	O0_N0_**Y1**	**489**	**376**	**279**	**148**
Putative secreted protein	GADI01006537 GANP01014738	**5**	20.1 (20.1)	**Y**	O0_**N1**_Y0	nd	nd	nd	nd
Venome MP3-like	GADI01008036 **IrSigP-37829_FR4_7-380**	**5**	10.8 (8.4)	**Y**	O0_N0_Y0	**0**	**0**	**0**	**443**
Secreted protein	**16939_FR1_1-184** **IrSigP-16941_FR1_1-451**	**4**	12.1 (12.1)	**Y**	**O4**_N0_Y0	**13**	**10**	**12**	**48**
Metis5	**1791_FR1_58-521** **IrSigP-245384_FR2_35-602**	**4**	4.8 (4.8)	**Y**	**O6**_N0_Y0	**791**	**731**	**422**	**476**
Uncharacterised protein	GADI01001927 GANP01013656	**4**	13.5 (8.8)	**Y**	**O2**_N0_Y0	nd	nd	nd	nd
Apyrase (5'-nucleotidase)	GADI01003062 **IrSigP-91535_FR1_1-605**	**4**	10 (7.9)	**Y**	O0_**N1**_Y0	**18**	**11**	**14**	**7**

Legend to the **Table 1**: Counts of assigned peptides to an individual protein were identified and quantified by the MaxQuant software engine. Proteins with a minimum of four assigned unique peptides and with zero assignment in the naïve sample are shown in the table. Only sequence coverages of more than 2% and Q values (representing the probability that the protein is a false hit) that equal zero are shown. Full proteomics assignment data are shown in the excel spreadsheet available as **Supplementary Data File**. Antigens present in the novel transcriptome of salivary glands from the I. ricinus ticks fed for six days are shown in bold, and their sequences are accessible through column “A” in the hyperlinked Source data 1 spreadsheet (see Material and Methods for download link).

a individual protein identified and quantified by the MaxQuant software engine.

b Normal font - accession number of the assigned protein in the GenBank; In bold - antigens present in the novel transcriptome of salivary glands from the I. ricinus ticks fed for six days. Sequences are accessible through column “A” in the hyperlinked Source data 1 spreadsheet (see Material and Methods for download link).

c Number of unique peptides used for the antigen identification.

d Sequence coverage.

e Signal peptide prediction using SignalP with >0.90 probability threshold (Almagro Armenteros et al., 2019). For Metis 5 metalloprotease, which lacks an N-terminus in our database, antarease I. scapularis homologue (XP_029834648.1; E value = 0; aa identity = 83%) was used for the SignalP prediction.

f Prediction of posttranslational modification. Numbers of O-glycosylation (O) and N-glycosylation (N) sites were predicted (> 0.75 prediction threshold) by NetOGlyc 4.0 and NetNGlyc 1.0, respectively (Steentoft et al., 2013). Y prediction of tyrosine sulfation (Chang et al., 2009) (threshold > 0.90).

g Average FPKM values in I. ricinus salivary glands transcriptomes (1D, 2D, 3D, 6D - days of feeding).

### Injection of Metalloprotease Inhibitor Impairs Tick Feeding *In Vivo* on a Rabbit but Not Feeding *In Vitro* on a Membrane

**Figure 4:**
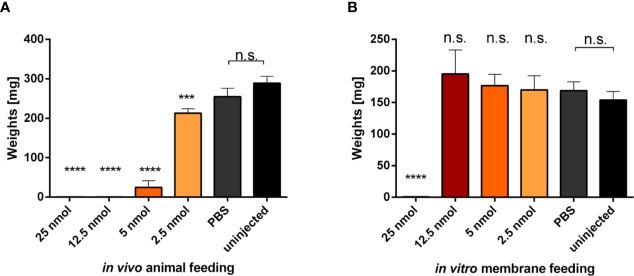
Evaluation of micro-injected phosphoramidon effect on tick feeding. Adult *I. ricinus* females were micro-injected indicated amounts of phosphoramidon solubilized in PBS. **(A)** Weights of engorged females fed on a rabbit. Bars represent the mean and SEM, n ≥25. **(B)** Weights of engorged females fed on an artificial membrane system. Data were obtained from two independent experiments. Bars represent a mean and SEM, n ≥12. Asterisks indicate statistical significance, *** indicate p = 0.0003, **** indicate p < 0.0001, n.s, not significant, when compared to the uninjected control.

To investigate the role of metalloproteases at the tick-host interface, we performed a comparative feeding on a rabbit (*in vivo*) and using an artificial membrane system (*in vitro*) with ticks that were microinjected with phosphoramidon, a potent naturally occurring inhibitor of neprilysin-like and ACE-like metalloproteases (Campbell, 2018). Ticks were micro-injected with different concentrations of phoshoramidon solution in PBS, ranging from 50 to 5 µg/µl, in a volume of 300 nl (25 to 2.5 nanomoles/tick injection). Ticks rested over-night before being placed for feeding. Ticks were then either put on a rabbit or on the membrane of an artificial feeding system. The highest concentration injected (50 µg/µl) was toxic and ticks died before feeding initiation or shortly thereafter, irrespective of the feeding mode (**Figure 4**). However, the lower concentrations (5–25 µg/µl), impaired the feeding of ticks on a rabbit, while ticks fed on a membrane managed to initiate and accomplish feeding until full engorgement (**Figure 4**). These data clearly show that tick salivary metalloproteases play a critical role in the feeding process on a living host.

## Discussion

Tick salivary gland transcriptomic (Perner et al., 2018) and proteomic (Narasimhan et al., 2007) repertoires wax and wane as functions of time during the initial period of tick feeding on a host. The salivary proteome repertoire also appeared to adjust according to the host species (Tirloni et al., 2017; Narasimhan et al., 2019). For increased authenticity in identifying peptide mass fingerprints, it is critical to have transcriptomic databases relevant to particular tick feeding stages and host type. We, therefore, assembled a salivary gland transcriptome from ticks fed on a rabbit for six days, i.e. the common time-point for pilocarpine-induced saliva collection, to match in time with proteomic pull-down and mass spectrometry. In this study, we identified and experimentally demonstrated that salivary enzymes and scavenging proteins play a critical role in interactions between the tick and host at the expense of immunological recognition during prolonged exposure to the host immune system. During this period, the host antibody response had sufficient time to develop. Antigens identified by repeated feeding of ticks thus have the potential to be harnessed for research into vaccination against tick feeding. Importantly, the recent study on guinea pigs and their acquired resistance to *I. scapularis* infestation revealed that tick salivary glycoproteins are the critical elicitors of tick resistance (Narasimhan et al., 2020). This finding agrees with our results showing that the majority of antigenic metalloproteases from *I. ricinus* saliva are predicted to be post-translationally modified by O-type and/or N-type glycosylation. If glycans need to be retained on the enzymes to induce protectivity, this needs to be taken into consideration in recombinant protein expression strategies for vaccination against ticks and tick-borne pathogens (Šimo et al., 2017).

Metalloproteases comprise the majority of coding sequences in the tick salivary gland transcriptomes, accounting for more than 5% of the total read of the entire transcriptome (Schwarz et al., 2013), as well as of salivary proteomes of various tick species, e.g. *Dermacentor andersoni* (Mudenda et al., 2014), *Haemaphysalis longicornis* (Tirloni et al., 2015), *Ixodes scapularis* (Kim et al., 2016), *A. americanum* (Kim et al., 2020). Despite their abundance, the physiological function of most metalloproteases in tick saliva remains unknown, although some were characterized as fibrinogenases (Francischetti et al., 2003). We can speculate about their additional possible interference with the host peptide signalling cascade of inflammatory responses or host matrix modulation. Our results show that some of the MA clan zinc-dependent metalloproteases of different families are the major immunogens present in the saliva of *I. ricinus* ticks and thus corroborate previous studies showing the recognition of metalloproteases by sera of repeatedly infested dogs and rabbits (Decrem et al., 2008) as well as by sera from people with an immune reaction to tick feeding (Becker et al., 2015). Vaccination trials with the metalloprotease Metis1 in rabbits led to decreased fecundity in *I. ricinus* ticks (Decrem et al., 2008), as well as with the metalloprotease BrRm-MP4 from *R. microplus* (Ali et al., 2015).

Three of five identified antigenic metalloproteases in *I. ricinus* saliva presumably act as endopeptidases, cleaving host (vertebrate) large protein or peptide substrates. M13-neprilysin-like metalloprotease is an endopeptidase and shares a 32% amino-acid identity with mammalian neprilysin, and contains a HEITH Zn-binding motif (Bland et al., 2008). Invertebrate M13 peptidases have been found in various organisms playing diverse roles from metamorphosis to immune responses (Bland et al., 2008). Neprilysin-like peptidases have undergone genome expansion and the *I. scapularis* genome encodes more than a hundred different isoforms (Gulia-Nuss et al., 2016). M2 Metallo-dipeptidyl carboxypeptidase (kininase) is a broad substrate specificity exopeptidase, containing an HEMGH Zn-binding motif, and sharing a 42% amino acid identity with insect and mammalian angiotensin-converting enzyme (ACE). However, it has been shown that the ACE-related protein from *R. microplus* does not cleave mammalian angiotensin as a substrate (Jarmey et al., 1995; Bastiani et al., 2002). Instead, the *I. scapularis* dipeptidyl carboxypeptidase was shown to exert a kininase activity, with the capacity to cleave host bradykinin, a key pro-inflammatory molecule (Ribeiro and Mather, 1998). The identified *I. ricinus* antigenic kininase is a likely homologue of the *I. scapularis* enzyme (XP_029826915.1; 90% amino acids identity over 99% coverage) and *R. microplus* ACE-like protein (54% amino acid identity over 95% sequence coverage), designated as Bm91 antigen (Riding et al., 1994). Vaccination with recombinant Bm91 produced in the eukaryotic *Pichia pastoris* system, however, did not confirm any effect against tick feeding or reproduction (Lambertz et al., 2012). When silenced, no significant deviations from feeding and reproduction in Bm91-RNAi ticks were observed either (Nijhof et al., 2007). M1 Puromycin-sensitive aminopeptidase is another exopeptidase, containing the HELAH Zn-binding motif, and shares a 44% amino acid identity with mammalian aminopeptidase N. The tick aminopeptidase N homologue has a strong predication for tyrosine sulfation. Such post-translational modification plays a critical role in its capacity to inhibit host haemostatic targets (Thompson et al., 2017). In insects, aminopeptidase N is mainly localized to the midgut membrane (Adang, 2004) and is a candidate molecule from the mosquito *Anopheles gambiae* for a *Plasmodium falciparum* transmission-blocking vaccine (Dinglasan et al., 2007). Interestingly, using specific rabbit polyclonal antibodies, nearly a 100% neutralisation of protease activity was achieved, which supports the concept of immunoglobulin-mediated neutralisation of proteases at the parasite-host interface (Dinglasan et al., 2007).

Besides the dominant metalloproteases, we also identified the enzyme apyrase as an immunogenic constituent of tick saliva. Apyrases hydrolyse host ADP and ATP, inhibiting key pathways of platelet and neutrophil aggregation and other pro-inflammatory reactions (Francischetti et al., 2009). Anti-apyrase immunoglobulins have been reported as markers of the sand fly *Lutzomyia longipalpis* bite, indicating an immunogenic nature of these enzymes, even in non-tick blood feeders (Milon et al., 2010). Vaccination of rabbits with *E.coli*-produced recombinant *Ornithodoros moubata* apyrase (GenBank: AGJ90350.1; 52% amino acid identity and 97% coverage with the *I. ricinus* immunogenic apyrase) led to a strong reduction in feeding in all developmental stages (Díaz-Martín et al., 2015), indicating a protective effect when used as a single-antigen vaccine.

Another natural *I. ricinus* antigen was identified as an apolipophorin-related molecule. Apolipophorins were originally described as important lipid transporters in insect hemolymph (Chino et al., 1981; Chino and Downer, 1982). Insect apolipophorins show a resemblance to tick vitellogenins and carrier proteins (Donohue et al., 2009), all of which are derived from a common ancestor and belong to the large lipid transfer protein superfamily (Smolenaars et al., 2007). This finding is not new, as homologous lipoproteins have previously been identified proteomically in pilocarpine-induced saliva of partially- and fully-fed *R. microplus* ticks (Tirloni et al., 2014), dopamine-induced saliva of partially-fed *D. andersoni* (Mudenda et al., 2014*)*, dopamine- and pilocarpine-induced saliva of *H. longicornis* nymphs and adults (Tirloni et al., 2015), pilocarpine-induced saliva of *I. scapularis* (Kim et al., 2016), *A. americanum* (Kim et al., 2020), as well as by antibody-mediated identification in phage display cDNA expression SG libraries from early timepoints of *A. americanum* feeding (Radulovic et al., 2014). Genetically tick-resistant bovines recognize more tick salivary proteins than sera of susceptible ones. Among *R. microplus* antigens identified by sera of resistant bovines, and not of susceptible ones, are apolipophorin and lipocalins (Garcia et al., 2017). The role of lipoproteins in tick saliva is currently unknown. The transcript encoding apolipophorin/vitellogenin (SigP-149886; GenBank: JAB71606.1) is predominantly expressed in the midgut rather than salivary glands in nymphs and adults of *I. ricinus* (Kotsyfakis et al., 2015), suggesting that it might not be a specific salivary secretory protein.

Using artificial membrane feeding of ticks, we demonstrated that the mere presence of anti-tick immunoglobulins in the blood meal had no significant effect on the progress of tick feeding. This can be explained by the fact that the processes by which antigens interact with the *in vivo* system are absent in the membrane feeding system. Such processes might include blood coagulation (ticks fed on defibrinated blood in the membrane feeding system), or leukocyte infiltration and degranulation. Host production of specific immunoglobulins may bring about neutralisation of the antigenic metalloprotease enzymatic activity. When fed repeatedly, potential antibody-mediated neutralisation of the proteases might lead to increased levels of peptidic inflammatory mediators and leukocyte infiltration, eventually causing rejection of ticks. Alternatively, as tick salivary Zn-metalloproteases were implicated in the prevention of fibrin clot formation and dissolution of the fibrin clots (Francischetti et al., 2003), we speculate that the ability of ticks exposed to protective immunoglobulins or a specific inhibitor to fully engorge in an *in vitro* membrane feeding system is due to the fact that ticks are served defibrinated blood. Any neutralisation of tick anti-clotting activity is, therefore, not physiologically penalized in this apparently “luxurious” feeding system.

## Conclusion

In this work, we have: 1) identified tick salivary proteins that are recognized by antibodies produced in a tick-immunized host (rabbit), using peptide mass fingerprinting; 2) assembled and made publicly available the tick salivary gland transcriptome of adult *I. ricinus* females from the 6th day of tick feeding; 3) demonstrated that neutralization of tick Zn-dependent metalloproteases by rabbit immunoglobulins or inhibition by a specific inhibitor prevent ticks from initiating and progressing through natural feeding but not artificial feeding.

We conclude that metalloproteases present in tick saliva target the components of the host hemostatic and defence systems that are absent during artificial membrane feeding. This explains why ticks feed successfully *in vitro* even in the presence of protective immunoglobulins or metalloprotease inhibitors.

## Data Availability Statement

The datasets presented in this study can be found in online repositories. The names of the repository/repositories and accession number(s) can be found in the article/**Supplementary Material**.

## Ethics Statement

The animal study was reviewed and approved by Central Committee for Animal Welfare, Czech Republic.

## Author Contributions

JP: conceptual design, membrane feeding of ticks and immunoprecipitation of antigens, wrote the manuscript draft. DH and PH: mass spectrometry. TH: phosphoramidon experiments and membrane feeding. SK: western blotting. JR: transcriptome assembly and edited the manuscript. PK: co-wrote and edited the manuscript. All authors contributed to the article and approved the submitted version.

## Funding

This study was funded mainly by the Czech Science Foundation (GACR) Grant No: 18-01832S to PK. JP and PK were additionally supported by the “Centre for research of pathogenicity and virulence of parasites” (no. CZ.02.1.01/0.0/0.0/16_019/0000759) funded by the European Regional Development Fund (ERDF) and Ministry of Education, Youth and Sport (MEYS).

## Conflict of Interest

The authors declare that the research was conducted in the absence of any commercial or financial relationships that could be construed as a potential conflict of interest.
