# Supplementary material for: The Central Role of Salivary Metalloproteases in Host Acquired Resistance to Tick Feeding
Source: Front Cell Infect Microbiol. 2020 Nov 18;10:563349. doi: 10.3389/fcimb.2020.563349 (PMC7708348; doi:10.3389/fcimb.2020.563349)
Supplement: Supplementary file 1 [file DataSheet_1.docx]

**The central role of salivary metalloproteases in the host acquired resistance to ticks feeding**

Perner Jan^1^, Helm Dominic^2^, Haberkant Per^2^, Hatalova Tereza^1^, Kropackova Sara^1^, Ribeiro Jose M^3^, Kopacek Petr^1^

*^1^ Institute of Parasitology, Biology Centre, Czech Academy of Sciences, 37005 Ceske Budejovice, Czechia*

*^2^ Proteomics Core Facility, The European Molecular Biology Laboratory (EMBL), Heidelberg, Germany*

*^3^ Laboratory of Malaria and Vector Research, National Institute of Allergy and Infectious Diseases, Bethesda MD, United States of America*

**Supplementary Figures**


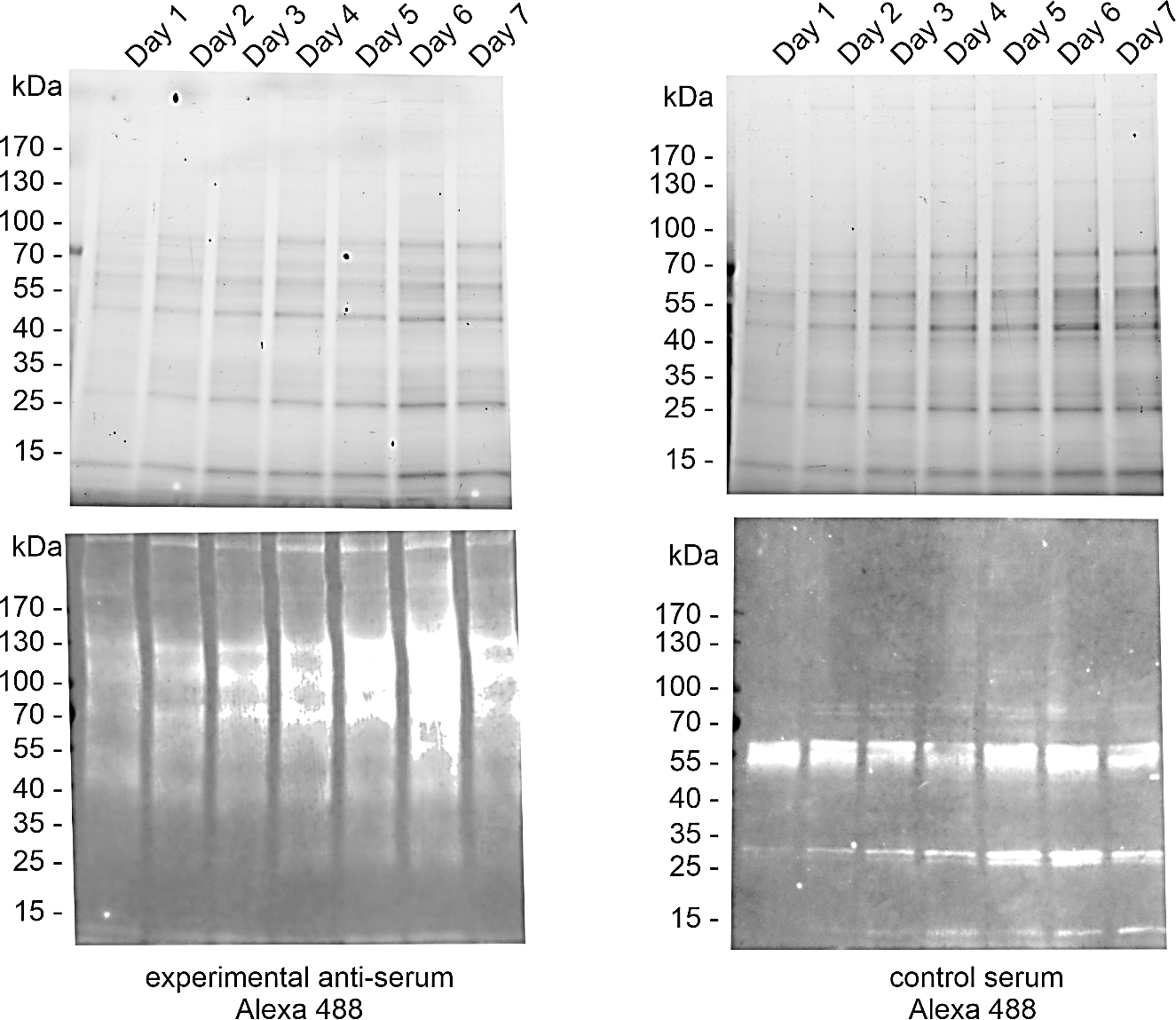


**Figure S1. Western blot evaluation of immunogenicity of tick midgut dissected during the course of feeding.** 10µg of midgut homogenates (PBS pH 7.2, 10μM E64, 0.1% Triton X100) were separated by reducing SDS PAGE in Criterion TGX Stain-Free polyacrylamide gel 4‒15% (BioRad); bands were visualised by the BioRad stain-free technology (upper images). Separated proteins were also transferred on a PVDF membrane for western blotting. Membranes were incubated with sera from a repeatedly-infested rabbit (experimental) or serum from a naïve rabbit (control) in dilution 1:1000. Signals were visualised through Alexa488-fused anti-rabbit secondary antibody (1:1000) in a ChemiDoc System (BioRad)

**
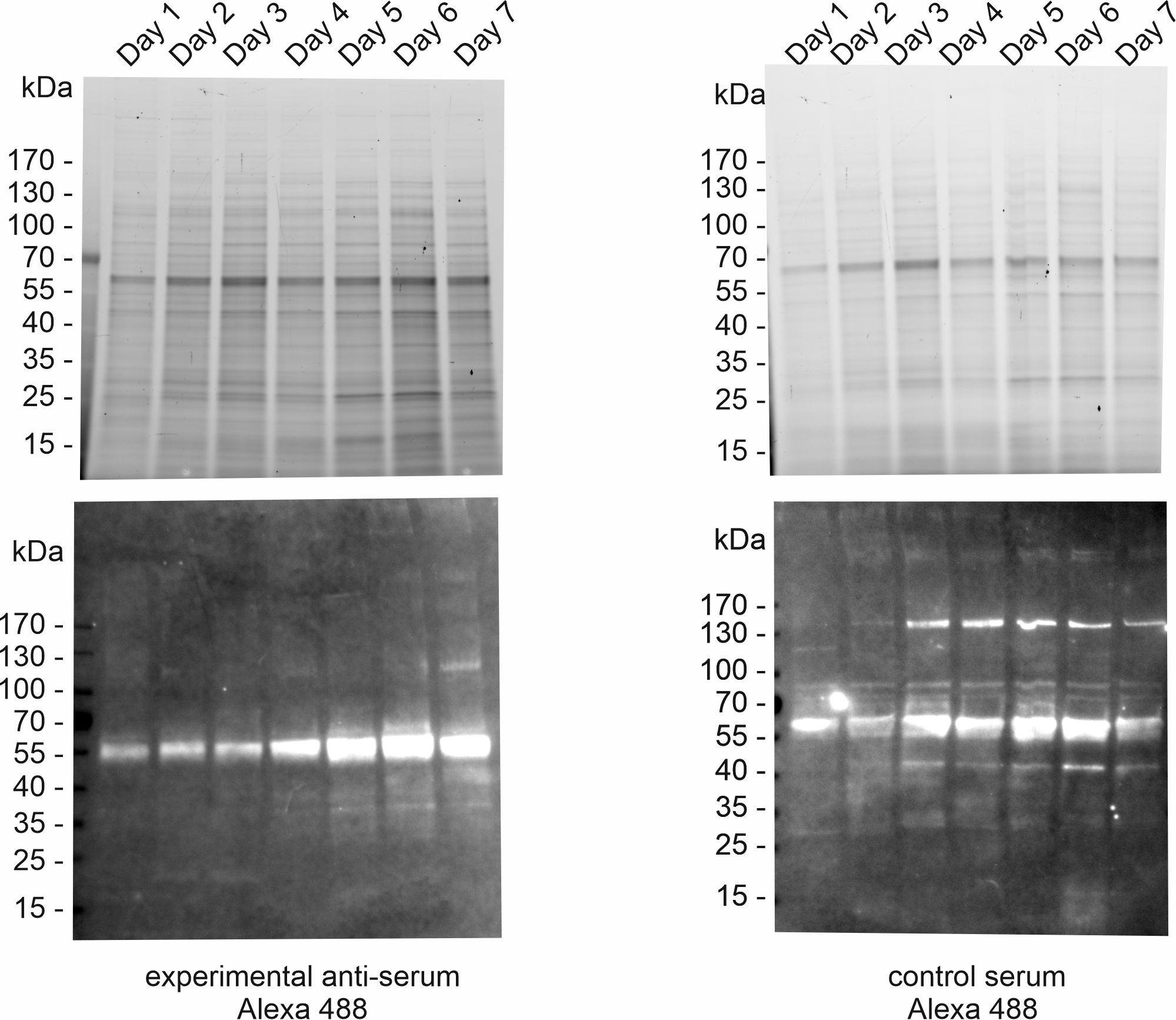
**

**Figure S2. Western blot evaluation of immunogenicity of tick salivary glands dissected during the course of feeding.** 10µg of salivary glands homogenates (PBS pH 7.2, 10μM E64, 0.1% Triton X100) were separated by reducing SDS PAGE in Criterion TGX Stain-Free polyacrylamide gel 4‒15% (BioRad); bands were visualised by the BioRad stain-free technology (upper images). Separated proteins were also transferred on a PVDF membrane for western blotting. Membranes were incubated with sera from a repeatedly-infested rabbit (experimental) or serum from a naïve rabbit (control) in dilution 1:1000. Signals were visualised through Alexa488-fused anti-rabbit secondary (1:1000) antibody in a ChemiDoc System (BioRad).

**
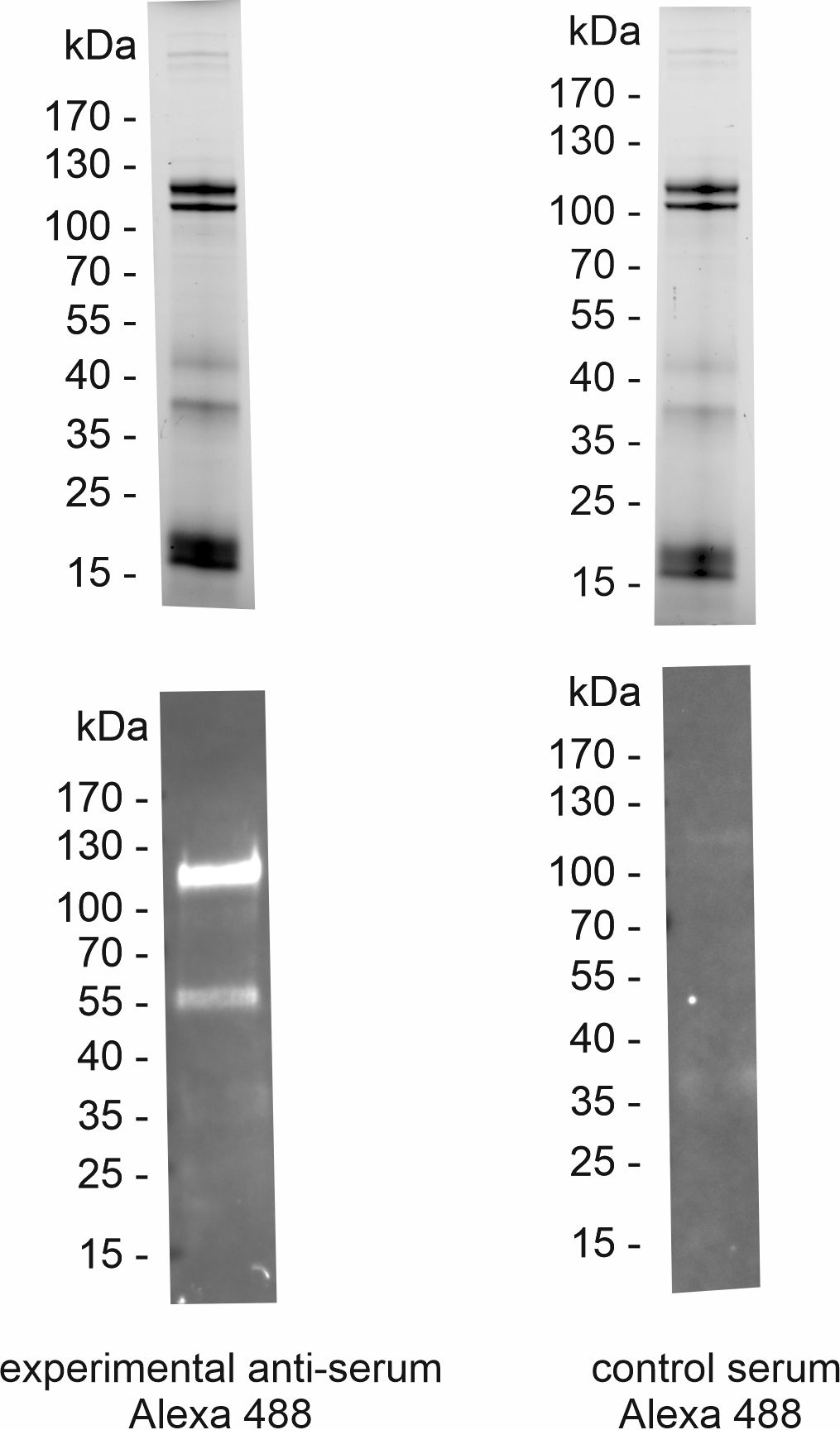
**

**Figure S3. Western blot evaluation of immunogenicity of tick saliva collected from partially-engorged adult females.** 10µg of pilocarpin-induced saliva was separated by reducing SDS PAGE in Criterion TGX Stain-Free polyacrylamide gel 4‒15% (BioRad); bands were visualised by the BioRad stain-free technology (upper images). Separated proteins were also transferred on a PVDF membrane for western blotting. Membranes were incubated with sera from a repeatedly-infested rabbit (experimental) or serum from a naive rabbit (control) in dilution 1:1000. Signals were visualised through Alexa488-fused anti-rabbit secondary antibody (1:1000) in a ChemiDoc System (BioRad).
